# Antipsychotic dose escalation as a trigger for Neuroleptic Malignant Syndrome (NMS): literature review and case series report

**DOI:** 10.1186/1471-244X-12-214

**Published:** 2012-11-29

**Authors:** Julie Langan, Daniel Martin, Polash Shajahan, Daniel J Smith

**Affiliations:** 1 Institute of Health an Wellbeing, Mental Health and Wellbeing, Gartnavel Royal Hospital, University of Glasgow, 1055 Great Western Road, Glasgow, G12 0XH, Scotland, UK; 2 NHS Lanarkshire, Greenmoss Community Health centre, University of Glasgow, Greenmoss Place, Bellshill, ML4 1PS, Scotland, UK

**Keywords:** Neuroleptic malignant syndrome, NMS, Rapid dose escalation, Rapid dose titration, Antipsychotics

## Abstract

**Background:**

“Neuroleptic malignant syndrome” (NMS) is a potentially fatal idiosyncratic reaction to any medication which affects the central dopaminergic system. Between 0.5% and 1% of patients exposed to antipsychotics develop the condition. Mortality rates may be as high as 55% and many risk factors have been reported. Although rapid escalation of antipsychotic dose is thought to be an important risk factor, to date it has not been the focus of a published case series or scientifically defined.

**Description:**

We aimed to identify cases of NMS and review risk factors for its development with a particular focus on rapid dose escalation in the 30 days prior to onset. A review of the literature on rapid dose escalation was undertaken and a pragmatic definition of “rapid dose escalation” was made. NMS cases were defined using DSM-IV criteria and systematically identified within a secondary care mental health service. A ratio of titration rate was calculated for each NMS patient and “rapid escalators” and “non rapid escalators” were compared. 13 cases of NMS were identified. A progressive mean dose increase 15 days prior to the confirmed episode of NMS was observed (241.7 mg/day during days 1–15 to 346.9 mg/day during days 16–30) and the mean ratio of dose escalation for NMS patients was 1.4. Rapid dose escalation was seen in 5/13 cases and non rapid escalators had markedly higher daily cumulative antipsychotic dose compared to rapid escalators.

**Conclusions:**

Rapid dose escalation occurred in less than half of this case series (n = 5, 38.5%), although there is currently no consensus on the precise definition of rapid dose escalation. Cumulative antipsychotic dose – alongside other known risk factors - may also be important in the development of NMS.

## Background

“Neuroleptic malignant syndrome” (NMS) derives from the French “syndrome malin des neuroleptiques” and was first described in 1960 by Delay [1] and colleagues in association with haloperidol. It is a potentially fatal idiosyncratic reaction to any medication which affects the central dopaminergic system, most commonly antipsychotics, with between 0.5% and 1% of patients exposed to these drugs developing the condition [2]. It is thought that all antipsychotics are capable of causing NMS, including the newer atypical agents, with case reports of clozapine [3], risperidone [4] and olanzapine [5] all causing NMS. Patients with suspected NMS usually have a history of anti-psychotic exposure; however this may not always be the case. The anti-emetic metoclopramide [6,7] the tricyclic antidepressant amoxapine [8], lithium [9] and phenelzine [10] have all been reported to cause NMS, presumably as a result of their dopamine blocking properties.

The precise pathophysiology of NMS remains unknown. It has been suggested that a marked and sudden reduction in central dopaminergic activity resulting from D_2_ receptor blockade within the nigrostriatal, hypothalamic, mesolimbic and mesocortical pathways may help to explain some of the clinical features of NMS such as rigidity, hyperthermia and altered mental state [11,12] This suggestion is supported by a number of factors including the observation that antipsychotic medication is the primary agent in most cases of NMS and the observation that NMS can also be induced by the abrupt withdrawal of dopamine.

However, D_2_ receptor antagonism does not fully explain all the signs and symptoms of NMS. In particular the occurrence of NMS with medications with low D_2_ affinity has led to the proposal that sympathoadrenal hyperactivity resulting from the removal of tonic inhibition within the sympathetic nervous system may play an important role in NMS [13]. This is supported by the frequent occurrence of autonomic symptoms in NMS, as well as demonstrated changes in urine and plasma catecholamine levels. Similarities with malignant hyperthermia have led to theories that a defect in calcium regulatory proteins within sympathetic neurons may be the key factor that triggers the onset of NMS [14]. Release of calcium from the sarcoplasmic reticulum of muscle cells has been shown to be increased with antipsychotic usage [15] and it may be that this could lead to the rigidity, muscle breakdown and hyperthermia seen in NMS.

Diagnosis of NMS is largely based on clinical history and the presence of specific clinical signs. Classically, NMS has been characterized by a triad of fever, rigidity and altered mental state [12]. However it is becoming increasingly clear that the presentation may be heterogeneous and this is reflected in the current DSM IV criteria (Table 1). Many conditions can mimic the presentation of NMS, including heat stroke, CNS infections, toxic encephalopathies, agitated delirium and more benign drug induced extra-pyramidal symptoms [12]. Given the clinical heterogeneity diagnostic uncertainty may occur. It is imperative that any underlying source of infection in particular is excluded and consequentially patients may be extensively investigated with serial blood and urine cultures, chest X ray, neuro-imaging and CSF analysis being obtained before underlying infections can confidently be excluded. A high level of clinician suspicion, vigilance and expertise is required in order to diagnose and initiate treatment of NMS promptly.

**Table 1:** DSM IV Research criteria for neuroleptic malignant syndrome

	
A.	Development of severe muscle rigidity and elevated temperature associated with the use of neuroleptic medication
B.	Two (or more) of the following
a.	diaphoresis,
b.	dysphagia,
c.	tremor,
d.	incontinence,
e.	changes in level of consciousness (ranging from confusion to coma),
f.	mutism,
g.	tachycardia,
h.	elevated or labile blood pressure,
i.	leukocytosis
j.	Laboratory evidence of muscle injury (e.g. elevated CPK creatinine phosphokinase).
C.	The symptoms in criteria A and B are not due to another substance, neurological or general medical condition
D.	The symptoms in A and B are no better accounted for by a mental disorder

A number of risk factors have been identified through several case control studies [16-22]. Rapid alteration and in particular escalation of anti-psychotic dose has emerged as an important risk factor for the development of NMS [16], with most cases occurring shortly after initial exposure [19]. NMS is less likely to occur in patients who have been stable on their dose of antipsychotic medication for a long time or who have good long-term compliance [20,23]. Antipsychotic polypharmacy, concomitant use of medications which predispose to NMS (including lithium) [24] and the use of intramuscular medication [21,22] all increase the risk of NMS. Other risk factors including agitation, dehydration, physical exhaustion, malnutrition, hyponatremia, thyrotoxicosis, alcohol or other psychoactive substance misuse [25] are all thought to be important in NMS. The presence of an organic brain syndrome or previous brain injury [20], poorly-controlled antipsychotic-induced extra pyramidal side effects (EPSEs) and iron deficiency [26] have also found to increase the risk of NMS. Males under 40 are often considered to be at greater risk of NMS but it is unclear if this is a reflection of increased use of antipsychotics within this population. Postpartum women may be at slightly elevated risk [27] and although family clustering [28] has been reported, there have been no studies to date investigating genetic vulnerability to NMS.

Rate of dose escalation of antipsychotic medication has been recognized as a risk factor for NMS [29]. Rate of patient titration onto a therapeutic dose is often multifactorial, with factors such as age, co-morbid physical health problems, previous antipsychotic exposure, history of side effects, severity of illness and the need for a rapid clinical response being important considerations [30]. Individual clinician preference and experience also plays a role. There are titration schedules available for certain medications, most notably quetiapine [31] and clozapine; however guidance is not available for all medications. In clinical practice there are occasions where antipsychotic dose is escalated more quickly than would be seen routinely. This practice is often termed “rapid dose escalation”. However there is no consensus on what rate of escalation would be defined as “rapid” and to date no mathematical calculation to quantify and compare such dose escalations has been considered.

NMS remains an uncommon, idiosyncratic and potentially fatal complication of antipsychotic medication. Despite the obvious role of antipsychotics in causing NMS, to our knowledge there have been no reports to date which specifically focus on patterns of antipsychotic dosing in the days leading up to an episode of NMS and no attempts to define “rapid dose escalation”.

## Construction and content

We aimed to systematically identify all cases of NMS within a defined UK health service population (NHS Lanarkshire, Scotland) and review potential risk factors with a focus on changes in antipsychotic dose in the 30 days prior to development of the syndrome. We compared this dosing to routine in-patient clozapine titration schedules and the dosing schedule for quetiapine. Based on our findings and review of the literature, we also aimed to define “rapid dose escalation”.

### Literature review

We reviewed the literature surrounding NMS using the most commonly searched databases including PubMed, Google scholar and Medline using keywords including “NMS” and “neuroleptic malignant syndrome”. We searched for a definition of rapid dose escalation using keywords “antipsychotic”, “neuroleptic”, “rapid dose escalation”, “rapid titration”, and “rapid increase”. We included case reports, case series, and systematic reviews.

### Systematic identification of NMS cases

This study was approved by local NHS research ethics committee and Research and Development committee (Ref 11/WS/0073). We conducted a retrospective analysis of all psychiatric contacts in a discrete geographical area in Scotland (NHS Lanarkshire, population 550,000) to systematically identify cases of NMS (recorded during the period 2002–2011). The electronic records assessed cover all secondary care psychiatric contacts and were phased into NHS Lanarkshire’s mental health service over the period 2002–2005 (the ‘Genysis’ database).

**Figure 1:**
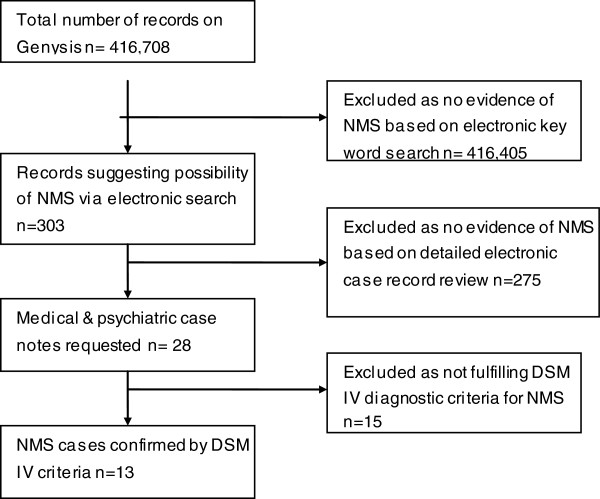
Identification procedure for NMS cases.

We searched for cases using the key words “neuroleptic malignant syndrome”, “NMS”, and “high CK”. Once possible cases were identified, the electronic record was initially reviewed by JL and DM. If there was a suggestion of NMS the psychiatric cases notes and, where appropriate, the general medical records were requested and reviewed independently by 2 psychiatrists (JL & DM) (Figure 1).

Cases of NMS were identified using DSM-IV criteria (Table 1), namely documented evidence of pyrexia and muscular rigidity in association with antipsychotic use. In the absence of both pyrexia and muscular rigidity, 2 or more of the following features were required to be documented in the notes - diaphoresis, dysphagia, tremor, incontinence, changes in level of consciousness (ranging from confusion to coma), mutism, tachycardia, elevated or labile blood pressure, leukocytosis or evidence of muscle injury (elevated CK). We required the symptoms seen not to be due to another substance, neurological or general medical condition. Any potential disagreement in diagnosis between authors was discussed and a consensus was reached.

Demographic details, information regarding predisposing risk factors for NMS and the episode of NMS were described and a detailed 30 day antipsychotic dose trajectory prior to the date of NMS onset was recorded. A cumulative antipsychotic dose was calculated using chlorpromazine equivalence to allow comparison and an estimation of total cumulative dose of antipsychotic (See Table 2). The antipsychotic dose trajectories generated were compared to the inpatient titration schedule for clozapine used in the UK and the quetiapine dosing schedules in the British National Formulary (BNF).

**Table 2:** Antipsychotic chlorpromazine equivalents

**Medication (mg)**	**Chlorpromazine Equivalence (mg)**
Chlorpromazine 100	100
Quetiapine 133.3	100
Amisulpiride 100	100
Olanzapine 5	100
Aripiprazole 7.5	100
Risperidone 2	100
Clozapine 200	100
Haloperidol 3	100
Sulpiride 200	100
ClopixolAccuphase (zuclopenthixol acetate) 100	100

The cumulative antipsychotic dose for each NMS patient was calculated for days 1 to 15 and days 16 to 30 and a ratio of ‘first 15 days’ to ‘second 15 days’ dose was generated. We then sub-categorized the NMS cohort into “rapid dose escalators” and “non rapid dose escalators” based on the ratio calculated. We defined rapid dose escalators as individuals who had at least a 4 times increase in their ratio of cumulative dose at days 16–30 compared to days 1–15. This conservative threshold was chosen pragmatically because a clozapine ratio of 3 during the first and second halves of a titration schedule is deemed standard practice and has a license to be titrated in this way. Quetiapine also has titration schedules recommended in the BNF [31] for both acute mania and schizophrenia and the ratio during the first and second halves of its titration schedule is 3.0 and 3.4 respectively. The two groups were then compared in terms of demographics, risk factors, clinical features and secondary complications.

## Discussion

We identified 13 cases of NMS: 7 were male (53.8%) and the average age was 46.2 years (range 19.4-70.7 years) (Table 3). The most common ICD 10 diagnosis was F20 Schizophrenia, schizotypal and delusional disorders diagnosis (n = 7, 53.8%) followed by F30 Mood (Affective) Disorders (2 n = 3, 3.1%). Other diagnoses included F0 Organic Mental Disorders (n = 2, 15.4%) and F60 Disorders of Adult Personality & Behaviour (n = 1, 7.7%). Additional clinical features of the NMS cases are outlined in Table 3.

**Table 3:** Clinical & demographic details

	**NMS patients (n = 13)**	**Cases 1–5 “rapid dose escalators” (n = 5)**	**Cases 6–13 “non rapid dose escalators” (n = 8)**
Mean age, years [95% C.I.]	46.2	47.8	45.2
Male, n (%)	7 (53.8)	2 (40)	5 (62.5)
ICD 10 Diagnosis			
F20 Schizophrenia n (%)	7 (53.8)	2 (40)	5 (62.5)
F30 Affective Disorders n (%)	3 (23.1)	2 (40)	1 (12.5)
F0 Organic Mental Disorder n (%)	2 (15.4)	0 (0)	2 (25)
F60 Disorder of Adult Personality & Behaviour n (%)	1 (7.7)	1 (20)	0 (0)
History of alcohol misuse, n (%)	5 (38.5)	2 (40)	3 (37.5)
History of substance misuse, n (%)	4 (30.8)	1 (20)	3 (37.5)
Compulsory Treatment, n (%)	5 (38.5)	2 (40)	3 (37.5)
History of non compliance, n (%)	9 (69.2)	3 (60)	6 (75)
Antipsychotic naïve, n (%)	1 (7.7)	1 (20)	0 (0)
History of extra pyramidal side effects, n (%)	8 (61.5)	2 (40)	6 (75)
Co prescribed oral antidepressant, n (%)	3 (23.1)	2 (40)	1 (12.5)
Co prescribed lithium, n (%)	2 (15.4)	1 (20)	1 (12.5)
Received IM medication, n (%)	5 (38.5)	2 (40)	3 (37.5)
Antipsychotic polypharmacy, n (%)	7 (53.8)	2 (40)	5 (62.5)
Required physical restraint, n (%)	5 (38.5)	2 (40)	3 (37.5)

In terms of diagnostic features of NMS, the most commonly seen features were raised CK (100% of cases where a CK was documented (n = 12)), altered GCS (n = 12, 92.3%), tachycardia (n = 11, 84.6%) and rigidity (n = 10, 76.9%). Pyrexia was documented in 5 cases (38.5%). Only 5 patients (38.5%) presented with the classic triad of rigidity, hyperthermia and altered conscious level (Table 4).

**Table 4:** Diagnostic features of NMS

**Documented diagnostic feature of NMS**	**All NMS patients (n = 4)**	**Cases 1–5 “rapid dose escalators” (n = 5)**	**Cases 6–13 “non rapid dose escalators” (n = 8)**
Pyrexia n (%)	5 (38.5)	1 (20)	4 (50)
Muscle Rigidity n (%)	10 (76.9)	3 (60)	7 (87.5)
Elevated CK n (%)	12 (100)^1^	4 (100)^1^	8 (100)
Mean CK U/l	2343.0	1672.5	2678.3
Altered GCS n (%)	12 (92.3)	4 (80)	8 (100)
Tachycardia n (%)	11 (84.6)	5 (100)	6 (75)
Mutism n (%)	7 (53.8)	2 (40)	5 (62.5)
Labile BP n (%)	6 (46.1)	1 (20)	5 (62.5)
Diaphoresis n (%)	8 (61.5)	2 (40)	6 (75)
Incontinence n (%)	6 (46.2)	1 (20)	5 (62.5)
Tremor n (%)	3 (23.1)	1 (20)	2 (25)

^1^information missing (diagnosed based on other criteria).

Those who were not considered “rapid dose escalators” were more likely to have BP instability, diaphoresis and incontinence compared to those who underwent “rapid dose escalation”. The mean CK value was higher in those who underwent standard dose titration and had high cumulative antipsychotic dose compared to those who underwent “rapid dose escalation” (2678.3U/l vs. 1687.2U/l) [Table 4].

All patients at minimum had a full physical examination undertaken to exclude underlying infection and had renal function, full blood count and inflammatory markers obtained. The majority of cases (n = 11, 84.6%) underwent neuro-imaging with 3 (23.1%) undergoing CSF examination.

All patients had their antipsychotic medication stopped when suspicion of NMS was raised by the clinical team and were commenced on IV fluids. 76.9% of patients (n = 10), required transfer to an acute medical ward where duration of stay varied from one to fifteen days. Two patients (15.4%) were treated with dantrolene, one (7.7%) with combination dantrolene and bromocriptine and one (7.7%) with ECT. Two patients (15.4%) had documented evidence of seizure activity during their episode of illness, one (7.7%) developed a DVT, one (7.7%) deranged LFTs and four (30.8%) developed acute renal failure.

### Medications

Table 5 describes in detail the medications received during the 30 days prior to NMS onset. Antipsychotic polypharmacy rates where high (7 cases, 53.8%), as was the use of parenteral medication (5 cases 38.5%).

**Table 5:** Antipsychotics prescribed within 30 days of NMS

**Patient**	**Antipsychotics prescribed within 30 days of NMS**
1	PRN Haloperidol
2	Risperidone & chlorpromazine
3	Olanzapine & PRN haloperidol
4	Quetiapine
5	Switched from clozapine to amisulpiride
6	Retitrated on clozapine & PRN haloperidol
7	Clozapine & amisulpiride combination treatment
	Purposeful overdose of clozapine. All antipsychotic stopped.
	Given sulpiride, haloperidol and accuphase
8	Olanzapine & PRN haloperidol
9	Switched from aripiprazole to risperidone & PRN chlorpromazine
10	Quetiapine & haloperidol
11	Risperidone
12	Quetiapine
13	Switched from olanzapine to amisulpiride

**Figure 2:**
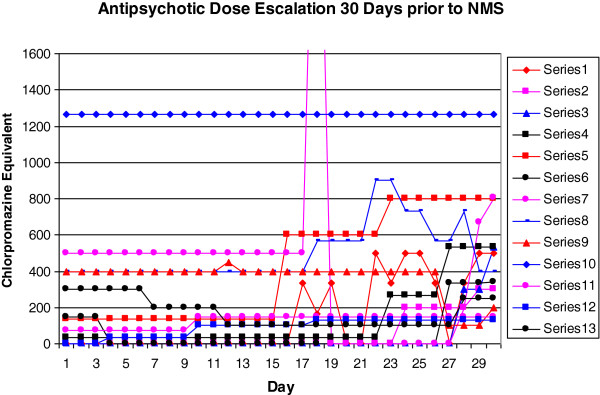
**30 day antipsychotic dose trajectory for NMS cases 1–13.** NB Patient 7 day 18 chlorpromazine equivalent peak is 3500 (off scale to allow ease of graph interpretation).

Figure 2 shows each individual’s antipsychotic dose trajectories 30 days prior to the onset of NMS. All antipsychotic dosages were calculated as chlorpromazine equivalents.

**Figure 3:**
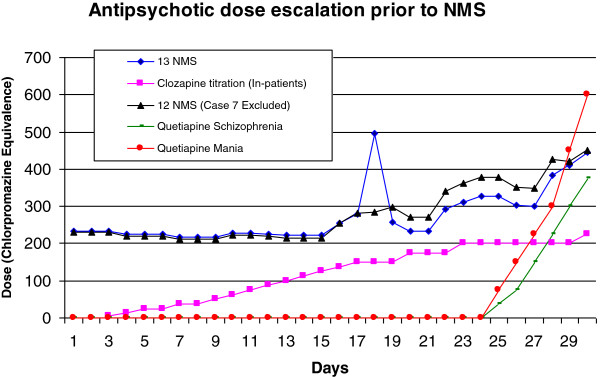
Mean 30 day antipsychotic dose trajectories.

Figure 3 shows the mean antipsychotic dose pattern for all 13 cases over the 30 days prior to NMS onset, the mean antipsychotic dose pattern minus patient 7 for the 30 days prior to NMS onset, the inpatient clozapine titration schedule and the quetiapine titrations schedules. In terms of patterns of prescribing, mean daily dose in the NMS cohort increased over the 30 day period- from 241.7 mg/day during days 1–15 to 346.9 mg/day during days 16–30. The mean ratio of dose escalation for NMS patients was 1.4.

In terms of “rapid dose escalation”, cases 1 to 5 received a greater than 4 times increase in their mean daily dose from days 1–15 (34.2 mg/day) compared to days 16–30 (281.3 mg/day). The mean cumulative dose for days 1–15 was 512.4 mg compared to 4219.9 mg for days 16–30.

For the “non rapid dose escalators”, the mean daily antipsychotic dose for days 1–15 and days 16–30 was markedly higher than that seen in both the “rapid dose escalator” cohort and the clozapine titration cohort. (406.8 mg/day vs. 34.2 mg/day vs. 63.8 mg/day during days 1–15 and 387.8 mg/day vs. 281.3 mg/day vs. 185.7 mg/day during days 16–30) (Table 6). The cumulative dose for the “non rapid dose escalators was also markedly higher (6102.5 mg for”non rapid escalators” vs. 512.4 mg for the “rapid escalators” vs. 893.8 mg for clozapine titrators in the first 15 days and 5817.2 mg for”non rapid escalators” vs. 4219.9 mg for the “rapid escalators” vs. 2600 mg for clozapine titrators during days 16–30) (Table 6).

**Table 6:** Mean antipsychotic dose prescribed

	**NMS patients (n = 13)**	**Cases 1–5 “rapid dose escalators” (n = 5)**	**Cases 6–13 “non rapid dose escalators” (n = 8)**	**Clozapine titration**
Mean cumulative total antipsychotic dose (mg) Days 1-15	3625.1	512.4	6102.5	893.8
Mean daily antipsychotic dose (mg) Days 1-15	241.7	34.2	406.8	63.8
Mean cumulative total antipsychotic dose (mg) Days 16-30	5202.8	4219.9	5817.2	2600
Mean daily antipsychotic dose (mg) Days 16-30	346.9	281.3	387.8	185.7
Mean Ratio (Days 16–30: Days 1–15)	1.4	7.3	1.1	2.9

### Risk factors for NMS

High titration rates of antipsychotics have been identified as a potential risk factor for the development of NMS and our study partially supports this observation. In our study, patients who developed NMS, a progressive mean dose increase 15 days prior to the confirmed diagnosis of NMS was observed. Considering NMS cases on an individual basis, not all received an increase in their antipsychotic dose. When compared to the known titration schedules, not all NMS patients were titrated at a higher rate - in fact only five patients (38.5%) were titrated at an elevated rate. In terms of cumulative antipsychotic dose received over the 30 day period, nine (69.2%) received a higher total dose compared to individuals being titrated on clozapine. Four patients (30.8%) received a lower total dose- of those receiving a lower cumulative dose; two were rapid escalators and two non rapid escalators.

### Rapid dose escalation

Rapid dose escalation, although a recognized clinical phenomenon, has not been systematically defined. There are case reports of rapid dose titrations occurring safely, however these involved small numbers of acutely unwell patients, being titrated on quetiapine [29] and olanzapine [30]. In these studies, “rapid dose escalation” was defined when patients received either a higher total dose of antipsychotic medication (olanzapine 30 mg- 40 mg or quetiapine 900 mg-1200 mg) or were more rapidly titrated onto quetiapine than is recommended by the BNF. However the titration rates of the patients included in the study were variable and in some antipsychotic polypharmacy occurred. No cumulative dose for patients was calculated and no mathematical attempt was made to quantify the “rapid dose escalation” described.

To date, our study is the only one available whereby an attempt to define rapid dose escalation mathematically based on comparison of cumulative dose over two time periods has occurred. This method allows comparison of dose escalation not only between different antipsychotics but also over different periods of time of titration. Provided doses are converted to chlorpromazine equivalents and cumulative doses over the first half of the titration schedule are compared to the second half, a ratio of dose escalation can be calculated. The individual dose escalation rate can then be compared to standard practice seen for clozapine and quetiapine allowing a more scientific evaluation of dose titration to be made. This method although limited by comparison only to standardised UK titration schedules, represented the best way to pragmatically define rapid dose escalation.

Our study suggests that a rapid rate of anti-psychotic dose escalation, although likely to be an important risk factor, is not universally observed in cases of NMS. Cumulative dose of antipsychotic may be equally important. It is worth noting that mean daily and mean cumulative antipsychotic doses were markedly higher both at day 15 and day 30 in the non rapidly escalating NMS cohort compared to the cohort who underwent rapid escalation. Individuals with high cumulative antipsychotic dose had a higher peak mean CK value during their episode of NMS.

### Diagnosis of NMS

The heterogeneous presentation of NMS is reflected in the current DSM IV criteria (Table 1), and our study supports observations of this. In our cohort, elevated CK, altered GCS and tachycardia were the most frequently documented features of NMS. Only five cases (38.5%), presented with the classic triad of rigidity, hyperthermia and altered conscious level. However given the entire clinical picture of our cases, their response to treatment combined with the fact 2 independent psychiatrists (JL & DM) reviewed the cases, we are confident that despite the heterogenic presentation they represent true NMS cases.

There have been some reports of NMS induced by second generation antipsychotics, having a propensity for atypical clinical presentations. However a recent comparison of NMS induced by first and second generation antipsychotics suggested that the clinical profile is “largely similar” [32]. It did recognize that that “clozapine induced NMS… (could be) differentiated by the relative lack of rigidity as a feature”. However interestingly in our cohort all patients who were exposed to clozapine in the 30 day period prior to their episode of NMS were noted to have rigidity as a feature of their presentation. This highlights the importance of a high level of clinician suspicion, vigilance and the importance of repeated review and reassessment in order to diagnose and initiate treatment of NMS promptly.

## Conclusions

Prescribers should be vigilant with regards to dose escalation of all psychotropic medications, particularly antipsychotics. Our study although limited by its small sample size and retrospective nature represents a pragmatic attempt to review antipsychotic dose escalation as a trigger for NMS. To our knowledge this is the only case series primarily focusing on antipsychotic dose escalation as a potential trigger for NMS and the first to attempt to define “rapid dose escalation” mathematically. The heterogeneity of the condition, combined with its rarity makes NMS difficult to research. Our case series reflects the complexity of the condition and by its retrospective nature allows it to be practicable to current clinical practice and adds to the limited evidence basis available.

Our findings indicate that it is not easy to predict what rate of antipsychotic dose escalation (if any) will result in the development of NMS. Indeed, the rate of dose escalation amongst less than half the cases (5 cases 38.5%) appeared relatively rapid, while in the others (8, 61.5%) was less than that seen in standard practice of titration with clozapine and quetiapine. Our findings also indicate that prescribers should also be mindful of cumulative antipsychotic dose and be aware of prescribing large doses of medication over prolonged periods of time.

It is clear that more work on both the heterogeneous presentation of NMS and its aetiology (both antipsychotic-related and other related factors) is warranted. A detailed review of a large series of patients with NMS (perhaps recruited internationally) matched to controls, with a particular focus on cumulative antipsychotic dose as well as rate of dose escalation, would be helpful in order to further enhance understanding of this condition. This may also allow a more scientific definition of “rapid dose escalation” to be determined.

## Abbreviation

NMS: Neuroleptic Malignant Syndrome.

## Competing interests

The authors declare that they have no competing interests.

## Authors’ contributions

Dr JL - 1st author & lead data collector. Dr DM - 2nd author & 2nd data collector. Dr PS - 3rd author, 3rd data collector & supervisory consultant. Dr DJS - 4th & corresponding author. All authors read and approved the final manuscript.

## Authors’ information

Julie Langan is a Clinical Lecturer working at the University of Glasgow and in NHS Greater Glasgow & Clyde.

Daniel Martin is a Research Fellow working at the University of Glasgow.

Polash Shajahan is a Consultant General Adult Psychiatrist working with NHS Lanarkshire. He has multiple research interests including psychopharmacology and individuals with high readmission rates. He is an honorary senior clinical lecturer at the University of Glasgow.

Daniel Smith is a reader in mental health and honorary consultant psychiatrist at the University of Glasgow. He has multiple research interests including bipolar illness and multimorbidity.

## Pre-publication history

The pre-publication history for this paper can be accessed here:

http://www.biomedcentral.com/1471-244X/12/214/prepub

## References

[B1] Delay J Pichot P Lemperier T Unneuroleptiquemajeur un phenothiazine et non resperine, l’haloperidol, dans le treatment des psychoses J Annales Medicos-Psychologiques 1960 118 145 152 13815606

[B2] Ananth J Parameswaran S Gunatilakem S Buroyne K Sidhom T Neuroleptic Malignant Syndrome and atypical anti-psychotic drugs Journal of Clinical Psychiatry 2004 65 464 470 10.4088/JCP.v65n0403 15119907

[B3] Sachdev P Kruck J Kneebone M Clozapine induced Neuroleptic malignant syndrome: review and report of new cases J Clin Psychopharmacol 1995 15 365 371 10.1097/00004714-199510000-00010 8830069

[B4] Dave M Two cases of risperidone induced neuroleptic malignant syndrome (ltr) Am J Psychiatry 1995 152 1233 1234 7542837 10.1176/ajp.152.8.1233b

[B5] Hall KL Taylor WH Ware MR Neuroleptic malignant syndrome due to olanzapine Psychopharmacol Bull 2001 35 49 54 12397878

[B6] Friedman LS Weinrauch LA D’Elia JA Metoclopramide induced Neuroleptic malignant syndrome Arch Intern Med 1987 147 1495 1497 10.1001/archinte.1987.00370080133023 3632154

[B7] Patterson JF Neuroleptic malignant syndrome associated with Metoclopramide Southern medical Journal 1988 81 674 675 10.1097/00007611-198805000-00035 3368821

[B8] Madakasira S Amoxapine induced neuroleptic malignant syndrome Drug Intelligence and Clinical Pharmacy 1989 23 50 61 10.1177/106002808902300111 2718483

[B9] Gill J Singh H Nugent K Acute lithium intoxication and neuroleptic malignant syndrome Pharmacotherapy 2003 23 811 815 10.1592/phco.23.6.811.32179 12820823

[B10] Heyland D Sauve M Neurloeptic malignant syndrome without the use of neurleptics Can Med Ass J 1991 145 817 819 1913410 PMC1335901

[B11] Bhanushali MJ Tuite PJ The evaluation and management of patients with Neuroleptic malignant syndrome Neurologic Clin 2004 22 389 441 10.1016/j.ncl.2003.12.006 15062519

[B12] Strawn JR Keck PE Caroff SN Neuroleptic malignant syndrome Am J Psychiatry 2007 164 870 876 10.1176/appi.ajp.164.6.870 17541044

[B13] Gurrera RJ Sympathoadrenalhyoactivity and the aetiology of neuroleptic malignant syndrome Am J Psychiatry 1999 156 169 180 9989551 10.1176/ajp.156.2.169

[B14] Gurrera RJ Is neuroleptic malignant syndrome a neurogenic form of malignant hyperthermia? Clin Neuropharmacology 2002 25 183 193 10.1097/00002826-200207000-00001 12151905

[B15] Adnet P Lestavel P Krivosic-Horber R Neuroleptic malignant syndrome British Journal of Anaesthetics 2000 85 129 135 10.1093/bja/85.1.129 10928001

[B16] Keek PE Pope HG Cohen BM McElroy SL Nierenberg AA Risk factors for neuroleptic malignant syndrome. Arch Gen Psychiatry 1989 46 914 9198 10.1001/archpsyc.1989.01810100056011 2572206

[B17] Bereardi D Amore M Keck PE Jr Troia M Dell’Atti M Clinical and pharmacological risk factors for neuroleptic malignant syndrome; A case control study Biology & Psychiatry 1998 44 748 754 10.1016/S0006-3223(97)00530-1 9798079

[B18] Berardi D Dell’Atti M Amore M De Ronchi D Ferrari G Clinical risk factors for neuroleptic malignant syndrome Human Psychopharmacology 2002 17 99 102 10.1002/hup.376 12404699

[B19] Caroff S Mann S Neuroleptic malignant syndrome Medical Clinics of North America 1993 77 185 202 8093494 10.1016/s0025-7125(16)30278-4

[B20] Pelonero AL Levebson JL Pandurangi AK Neuroleptic malignant syndrome; a review PsychiatrServ 1998 49 1163 1172 10.1176/ps.49.9.1163 9735957

[B21] Sachdev P Mason C Hadzi-Pavlovic D Case control study of neuroleptic malignant syndrome Am J Psychiatry 1997 154 1156 1158 9247408 10.1176/ajp.154.8.1156

[B22] Viejo LF Morales V Punal P Perez JL Sancho RA Risk factors in neuroleptic malignant syndrome a case control study ActaPsychiatrica Scandinavia 2003 107 45 49 10.1034/j.1600-0447.2003.02385.x 12558541

[B23] Berman BD Neuroleptic malignant syndrome: a review for neurohospitalists The Neurohospitalist 2011 1 41 47 10.1177/1941875210386491 PMC3726098 23983836

[B24] Buckley PF Hutchinson M Neuroleptic malignant syndrome J NeurolNeurosurg Psychiatry 1995 58 271 273 10.1136/jnnp.58.3.271 PMC1073359 7897404

[B25] Itoh H Ohtuska N Ogita K Malignant Neuroleptic syndrome: its present status in Japan and clinical problems FloiaPsychiatricaetNeurologica Japonica 1977 31 565 576 608659

[B26] Rosebush PI Mazurek MF Neuroleptic malignant syndrome: a review Lancet 1998 49 149 151 10.1016/0140-6736(91)90138-f 1677067

[B27] Alexander PJ Thomas RM Das A Is risk of neuroleptic malignant syndrome increased in the postpartum period? Journal of Clinical Psychiatry 1998 59 254 255 10.4088/JCP.v59n0509a 9632037

[B28] Otani K Horiuchi M Kondo T Is the predisposition to neuroleptic malignant syndrome genetically transmitted? British Journal of Psychiatry 1991 158 850 853 10.1192/bjp.158.6.850 1678666

[B29] Pajonk F-GB Schwertner AK Seelig MA Rapid dose titration of quetiapine for the treatment of acute schizophrenia and acute mania: A case series J Psychopharmacol 2006 20 119 124 16204326 10.1177/0269881105056665

[B30] Baker RW Kinon BJ Maguire GA Liu H Hill AL Effectiveness of rapid initial dose escalation of up to forty milligrams per day of oral olanzapine in acute agitation J Clin Psychopharmacol 2003 23 342 348 10.1097/01.jcp.0000085406.08426.a8 12920409

[B31] BNF 63 March 2012 4.2.1 Antipsychotic drugs 235 http://www.medicinescomplete.com/mc/bnf/current/PHP2284-quetiapine.htm

[B32] Trollor JN Chen X Chitty K Sachdev P Comparison of neuroleptic malignant syndrome induced by first and second generation antipsychotics BJPsych 2012 201 52 56 10.1192/bjp.bp.111.105189 22626633

